# Vapor Phase Ammonia Curing to Improve the Mechanical Properties of Antireflection Optical Coatings Designed for Power Laser Optics

**DOI:** 10.3390/gels9020140

**Published:** 2023-02-07

**Authors:** Jérémy Avice, Guillaume Brotons, Pascal Ruello, Gwenaëlle Vaudel, Amira Guediche, Hervé Piombini

**Affiliations:** 1 CEA, DAM Le Ripault, 37260 Monts, France; 2 Institut des Molécules et Matériaux du Mans, UMR 6283 CNRS-Le Mans Université, Av. Olivier Messiaen, CEDEX 9, 72085 Le Mans, France

**Keywords:** sol–gel, ammonia curing, infrared analysis, optical index, shrinkage

## Abstract

Projects of inertial confinement fusion using lasers need numerous optical components whose coatings allow the increase in their transmission and their resistance to high laser fluence. A coating process based on the self-assembly of sol–gel silica nanoparticles and a post-treatment with ammonia vapor over the surfaces of the optical components (“ammonia curing process”) was developed and successfully optimized for industrial production. Manufacturing such antireflective coatings has clear advantages: (i) it is much cheaper than conventional top-down processes; (ii) it is well adapted to large-sized optical components and large-scale production; and (iii) it gives low optical losses in transmission and high resistances to laser fluence. The post-treatment was achieved by a simple exposition of optical components to room-temperature ammonia vapors. The resulting curing process induced strong optical and mechanical changes at the interface and was revealed to be of paramount importance since it reinforced the adhesion and abrasion resistance of the components so that the optical components could be handled easily. Here, we discuss how such coatings were characterized and how the initial thin nanoparticle film was transformed from a brittle film to a resistant coating from the ammonia curing process.

## 1. Introduction

The research into renewable sources of energy production is an important challenge for the international community in order to overcome the decrease in oil resources throughout the world. In addition to green energy, two great research projects are currently being developed: the magnetic confinement within the ITER facility [1,2] and the inertial confinement within the NIF [3], the LMJ [4], and the SG-IV [5], projects. This paper presents the new advances in the development of the optical components of the Laser MégaJoule (LMJ).

This laser is currently being built in France for the simulation program of the Commissariat à l’Energie Atomique et aux Energies Alternatives (CEA). The LMJ has already demonstrated that it fits all the specifications required to begin experiments. It is based on the focusing of 176 laser beams, providing 1.4 MJ energy (400 TW) at 351 nm with a 3 ns pulse duration on a millimetric target [3,6]. These one hundred and seventy-six similar laser beams are grouped into twenty-two bundles; each bundle is divided into two quads of four laser beams. Each laser channel is composed of the same group of large optical components with a 400 × 400 mm^2^ size (mirrors, lenses, frequency converters, laser glasses, gratings, etc.) that are used to amplify and carry the beam from the oscillators up to the target placed at the center of the experiment chamber. Each optical component is coated to increase the energetic efficiency of the laser. More particularly, the components working in transmission are coated with an antireflective layer made of silica nanoparticles that were manufactured by the sol–gel process following the well-known Stöber method [7]. In this process, the antireflective layers are deposited with the dip coating technique on both sides at the same time with similar thicknesses.

After coating, the transmission reaches a value close to 100%, and the parasite reflections into the upstream components of the laser channel are minimized. The refractive index, n_L_, of a material deposited by the sol–gel process can be matched by controlling the porosity in order to obtain an index near the square root of the substrate refractive index, n_S_. As the lenses are made of fused silica with n_S_ = 1.45 as its refractive index, colloidal silica, with its refractive index n_L_ = 1.22, has the ideal refractive index to make an antireflective coating on a silica substrate. The thickness of this layer, t, is matched according to the antireflective wavelength, λ_0_. Indeed, for an antireflective layer, the optical thickness (n_L_.t) must be equal to λ_0_/4, i.e., either t = 216 nm for an antireflective film at λ_0_ = 1053 nm or t = 72 nm for λ_0_ = 351 nm. Layers with a 216 nm thickness are called AR 1ω, and layers with a 72 nm thickness are called AR 3ω. These layers are manufactured in one step without heating. In addition, it is impossible to manufacture such antireflective coatings from PVD (physical vapor deposition) techniques with only one layer because there is no material with an index as low as this, with the exception of the Glancing Angle Deposition technique (GLAD [8]), which is limited to small-sized components. Furthermore, antireflective coatings are known to have a high laser damage threshold [9,10,11,12,13,14]. In the case of laser damage occurring during the lifetime of a sol–gel optic, it is easy to remove the coating and coat it again afterward without the need for polishing steps. This is a great asset for maintaining low maintenance costs. Nevertheless, the sol–gel layers are deposited at room temperature with weak adherence and abrasion resistances, and the handling of such components after coating is a difficult engineering challenge.

To increase their mechanical resistance, a post-treatment using ammonia vapor is subsequently carried out. This room temperature process induces a mechanical strengthening of the layer while maintaining a good resistance under a laser flux as well as good optical properties [14,15,16]. The laser-induced damage thresholds (LIDTs) for layers that were subjected to several curing times have already been published [14]. In our experimental conditions (in the mode R/1 at 355 nm with an equivalent duration pulse τ_equ_ = 11.9 ns and a beam diameter of ϕ_FWHM_ = 92 µm), for layers with a thickness of 220 nm, the LIDTs were nearly 70 J·cm^−2^. This process is now being carried out in industrial conditions and at large-scale production rates [17]. In this paper, we studied nanoscale interfacial physicochemical modifications due to the ammonia curing process, and we discussed the optimization that allowed the process to be transferred to an industrial scale. Subtle changes from the post-treatment were observed such as shrinkage of the layer, which had to be taken into account to reach the desired thickness; changes in the refractive index and extinction coefficient, which were linked to an increase in the porosity; a layer crazing; an increase in the elastic modulus due to the Van der Waals bonds that were modified into covalent or hydrogen bonds; and a change in the coating contact angle with water, which gave a hydrophobic character to the layer and thus reduced the organic contamination [18,19]. The recent use of several analytical spectroscopies and other methodologies corroborated the excellent results obtained on a larger-scale investigation. The control of the resulting coating process was subsequently achieved in terms of the physical properties and reproducibility (index, thickness, curing time, etc.) as well as in terms of the optimization of its duration. Therefore, we minimized its production and maintenance costs.

## 2. Results and Discussion

All laboratory experiments presented here were carried out on polished silica substrates or on silicon wafers with a diameter of 50 mm. The LMJ optical components were square, and their sizes were 400 × 400 mm^2^.

### 2.1. Chemical Modifications during Ammonia Curing

**Figure 1:**
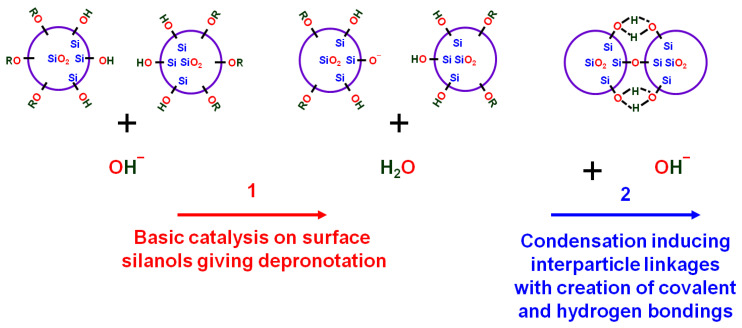
Schematic representation of the chemical evolution of ammonia curing.

**Figure 2:**
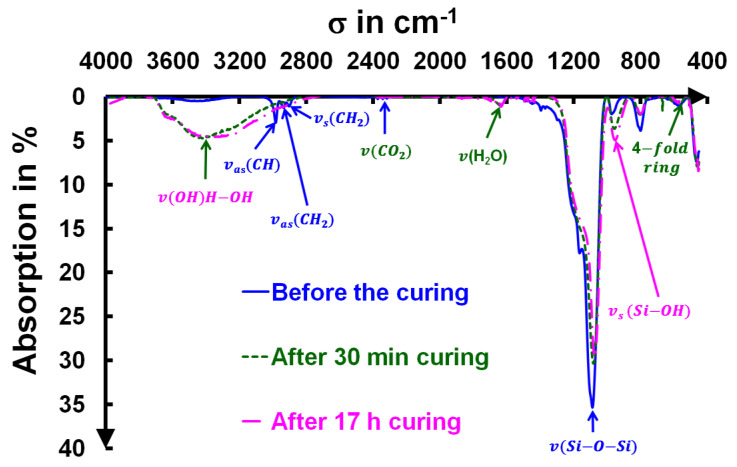
Evolution of IR spectra before and after different curing times (30 min and 17 h) for the same film.

**Figure 3:**
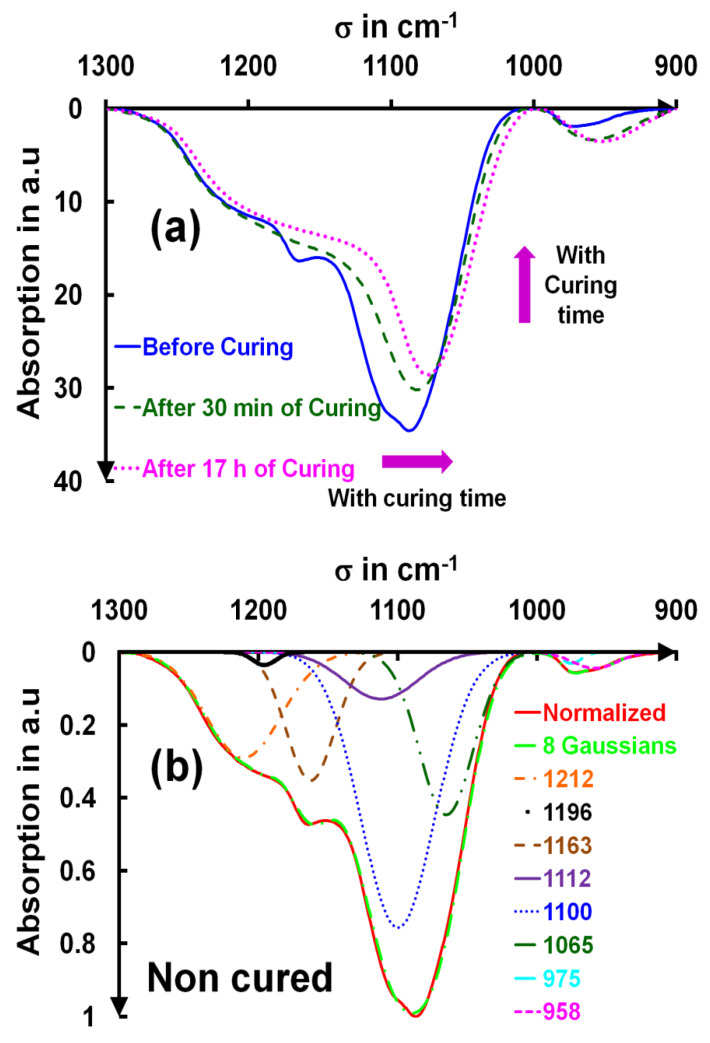

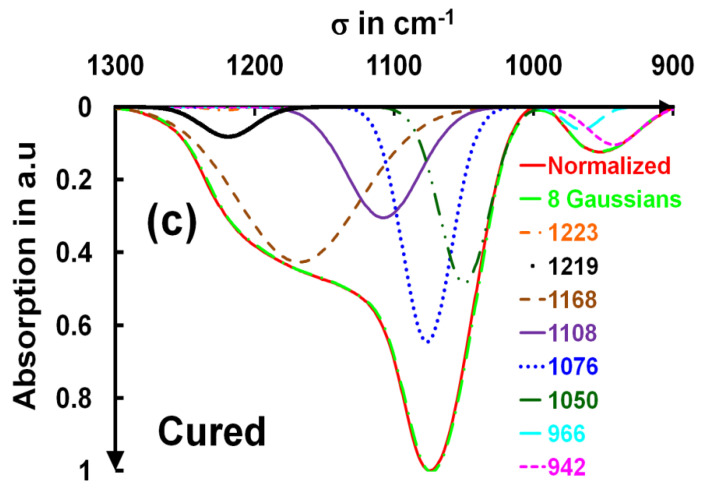
FTIR spectra of NH_3_-cured and uncured of the main Si–O–Si peak (**a**), decomposition of the Si–O–Si band into eight Gaussian contributions for a non-cured layer (**b**), and a cured layer for 17 h (**c**).

The observed shrinkage and the refractive index evolution promoted by the ammonia treatment can be explained by the surface hydrolysis of the ethoxy groups initially covering the surface of the silica nanoparticles (catalyzed by the nucleophilic NH_3_ and/or OH^−^ groups contained in the water that condensed inside the mesoporosity of the films) followed by the sol–gel condensation of the silanolate and silanol groups. The successive formation of siloxane bonds also favored the creation of the hydrogen bonds of the vicinal silanols, reducing the average distance between the silica nanoparticles. A typical sketch of this chemical process is provided in **Figure 1**, and, thanks to the infrared (IR) spectroscopy measurements, all these successive processes were described at the molecular level and compared to previous investigations [14,15,16,20]. A precise deconvolution of each spectral component was carried out as discussed later on (**Figure 2** and **Figure 3**).

To check for chemical modifications during the ammonia curing, a 300 nm thick layer was measured using IR spectrophotometry at selected curing times. More than 25 measurements were made over 50 h. Several transmission spectra are plotted in **Figure 2** corresponding to the same sample (uncured, 30 min, and 17 h of curing). The chemical modifications were fast; after only 30 min of curing, the infrared spectrum was close to that acquired after 17 h of curing.

The ammonia curing treatment affected the surface chemistry of the SiO_2_ colloids, with clear modifications of the film’s IR spectra, as shown in **Figure 2**. After thirty minutes of curing, the film was stabilized, and only weak chemical changes could be seen in the Fourier transform infrared (FTIR) spectroscopy measurements. It appeared that the hydrolysis kinetics were faster than the shrinkage phenomena. The number of silanol bonds and hydrogen bonds appeared to be stabilized after 30 min. The presence of CH_2_ and CH_3_ groups, evidenced by the uncured layer, came from the ethoxy groups (and possible residues of ethanol that was used for the preparation of the layer) that had not yet been removed from the layer during the initial measurement. They disappeared after curing. Their hydrolysis produced some water in the silica and resulted in a more hydrophilic surface capable of absorbing water (visible at 1630 cm^−1^) [21,22]. However, a change in the silica network was still observed over time, as shown subsequently. Before this, we described the general assignment of the IR bands. The analysis of such silica infrared data has been previously reported by Innocenzi et al. [23].

The shapes of the absorption bands at 970 cm^−1^ and 1090 cm^−1^ were analyzed in terms of multiple Gaussian components (FTIR spectra of **Figure 3a**), and they provided further insight into the mechanism of curing. The best decomposition of these absorbance bands into elementary Gaussian contributions was obtained with six Gaussians for siloxane peaks according to Lisovskii et al. [24] and Innocenzi et al. [23,25]. Two Gaussians were necessary to reproduce the silanol peaks over the 900–1300 cm^−1^ range. Before the decomposition, both baselines of the Si–OH and S–O–Si peaks were subtracted. The experimental curves were adjusted using **Equation (1)**:

(1) $${\mathrm{Abs}}_{\mathrm{Fit}}\left(\sigma \right)=\overset{i=2\;\mathrm{or}\;6}{\underset{i=1}{\sum }}A_{i}e^{-\frac{1}{2}\frac{{\left(\sigma \;-{\;\sigma }_{i}\right)}^{2}}{\omega_{i}^{2}}}$$

where A_i_ is the Gaussian amplitude, σ_i_ is the spectral position and ω_i_^2^ is the variance of the Gaussian contribution. The A_i_, σ_i_, and ω_i_^2^ values were determined by the minimization of the R^2^ factor defined by **Equation (2)**:

(2) $$R^{2}=\overset{\sigma =\sigma_{2}=1000\;\mathrm{or}\;1300}{\underset{\sigma =\sigma_{1}=900\;\mathrm{or}\;1000}{\sum }}{\left({\mathrm{Abs}}_{\mathrm{Normalized}\;\mathrm{Measurement}}\left(\sigma \right)-{\mathrm{Abs}}_{\mathrm{Fit}}\left(\sigma \right)\right)}^{2}$$

where Abs_Normalized Measurement_ is a measured spectrum, which was normalized to 1.

The optimization was also conducted over a narrow spectral range [1000–1300 cm^−1^]. In this way, we could see how much the contributing bands changed upon curing. The results are given for a non-cured layer and a layer cured for 17 h in **Figure 3b,c**.

**Figure 4:**
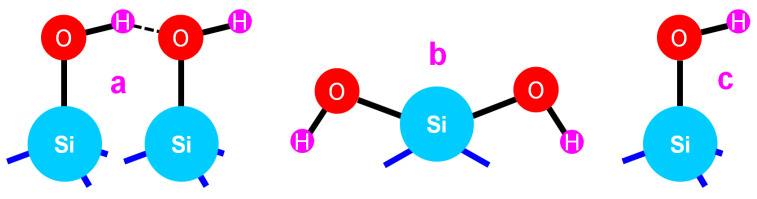
Different sorts of silanols: (**a**) vicinal silanols, (**b**) geminal silanols, and (**c**) isolated silanols (not found here).

A complete attribution of the absorption bands was performed for the uncured and cured materials. For both types of layer, there were several absorption peaks of siloxane groups at 1080 cm^−1^ (TO3 mode ν_as_ Si–O–Si: transverse optical antisymmetric stretching), at 800 cm^−1^ (TO2 mode ν_s_ Si–O–Si: transverse optical symmetric stretching of the O atom along a line bisecting the Si–O–Si angle) and 460 cm^−1^ (TO1 mode ρ Si–O–Si: transverse-optical rocking, i.e., motions perpendicular to Si–O–Si, of the oxygen bridging two adjacent Si atoms) [21,25]. A broad band between 2800 and 3800 cm^−1^ corresponding to the stretching bonds (ν_s_ H–O–H around 3300 cm^−1^) and vicinal and geminal silanol groups were also evidenced (**Figure 4a,b**). No isolated silanol group (**Figure 4c**) was detected at 3740 cm^−1^ [15]. The stretching bonds at 2983 cm^−1^ (ν_s_ CH_3_), 2938 cm^−1^ (ν_as_ CH_2_), and 2907cm^−1^ (ν_s_ CH_2_), corresponding to the CH_3_ and CH_2_ of the ethanol [22,26,27] or ethoxy groups, disappeared after the ammonia curing. We also observed a shift in the sharp peak shoulder corresponding to a Si–O antisymmetric stretching bond (1090 cm^−1^) [21,23,24] towards a low wavenumber, and its intensity decreased. An increase at 970 cm^−1^ of the silanol stretching vibration bonds (ν_s_ Si–OH) after hydrolysis was noticed [22,23,26]. The ammonia curing produced a decrease in the absorption at 800 cm^−1^ and an increase in the absorption at 1630 cm^−1^ corresponding to the ν_H2O_ bond in the silica [21,22]. The absorption at 575 cm^−1^ indicated a silica structure with four-fold siloxane rings [23,24,27,28,29,30]. Residual traces of CO_2_ were observed at around 2340 cm^−1^.

The ammonia curing was carried out in a basic atmosphere (pH > 10), and the condensation of the siloxane bonds competed with their re-dissolving [31]. In the presence of nucleophilic agents, this water interacted with the siloxane bonds (1080 cm^−1^ and 800 cm^−1^) to produce vicinal silanols (970 cm^−1^ and 2800–3800 cm^−1^), leading to an increase in the silanol absorption band and a slight decrease in the siloxane absorption band. These chemical modifications are very likely to alter colloid–colloid interactions, changing from Van der Waals to hydrogen and covalent bonds. Such changes induced contractions of the colloidal network leading to a shrinkage in the layer thickness and an increase in the elastic modulus as will be discussed in the next sections [15]. In such a situation, structural stress is expected to increase and can lead to cracks consistent with previous observations [15]. This strain in the silica layer was verified by the shift in the absorption peak of the Si–O–Si [28] (the TO and LO bonds moved towards the low wavenumbers). This shift was consistent with the decrease in the refractive index [28,30,32], which was confirmed by the spectrophotometric measurements and their interpretation, as described in Section 2.2. The decrease in the absorption Si–O–Si peak and the reduction in its absorption coefficient α_d_ were assigned to an increase in the porosity [25,33]. Although the films shrank in thickness by more than 17% during the curing, the refractive index decreased. This unambiguously revealed a loss of matter, which corresponded to the evaporation of the ethanol and water molecules trapped in the porosity, which were released from the hydrolysis and condensation reactions between the colloids.

**Figure 5:**
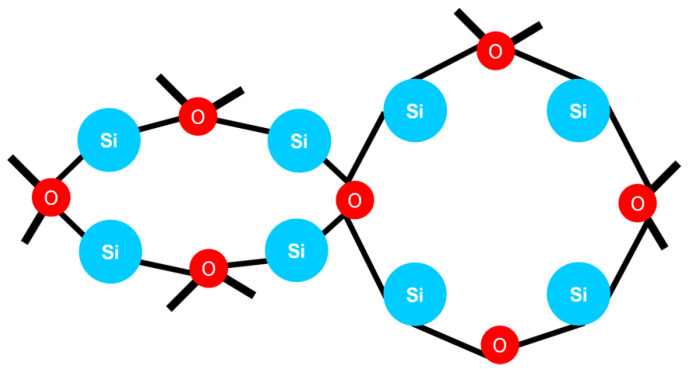
Diagram of the four-fold siloxane rings in sol–gel silica.

The first decomposition showed two absorption peaks [21,22,23,24,25,27,28] at 958 and 975 cm^−1^ for the uncured layers, which moved after curing to 942 and 966 cm^−1^. These peaks were associated with the stretches of Si–O– and Si–OH groups, respectively [23,27,31]. The peak order for the second decomposition followed the order given by Innocenzi et al. These decompositions are still controversial [23]. Therefore, this global fit allowed us to reach an unprecedentedly precise assignment of the IR peaks for the uncured and cured ammonia layers respectively. The absorptions at 1065 (uncured) and 1050 cm^−1^ (cured) were assigned to the TO3 mode, those at 1100 and 1076 cm^−1^ were assigned to the LO4 mode, those at 1112 cm^−1^ and 1108 cm^−1^ were assigned to 4 f-TO [27,28] (**Figure 5**), those at 1163 and 1168 cm^−1^ were assigned to the LO3 mode; those at 1196 and 1219 cm^−1^ were assigned to TO4 mode, and lastly, those at 1212 and 1223 were assigned to 4f-LO.

The discrepancy between the achieved fits leaving various parameters free, and the fits where the bonding corresponded to Innocenzi’s bonds are provided in **Table 1**. The positions of these vibration modes (TO3, LO3, and LO4) changed with the film thickness: the thicker the layer, the more the peaks shifted towards the lower wavenumber [34].

From the ammonia curing, the peak positions corresponding to the ν_Si-O_-mode increased due to the broken Si–O bonds coming from the four-fold siloxane rings [28] that were predominant after curing. The TO3 and LO4 modes moved after curing towards a low wavenumber and this suggested a porosity increase and thus a decrease in the refractive index [23,30]. A porosity increase was also seen through the increase in the area of the LO3 mode [23,30]. The area and the amplitude of the LO4 mode strongly decreased, which suggested a change to a more ordered structure [23] and the TO4 mode became stronger. All these findings were consistent with our hypothesis sketched in **Figure 1** and with the mesoscopic rearrangement evinced in the atomic force microscope (AFM) studies, which are presented below. The behavior of this post-process was similar to the effect of thermal annealing in mesoporous silica [23], pointing to a stabilization of the films (total surface minimization with matter rearrangement and/or silica colloid densification), which also induced an increase in the mechanical properties. The structure order change was also observed by the amplification of the 4f-TO mode and a reduction in the 4f-LO mode after curing.

### 2.2. Chemical Spectral Modifications Due to Ammonia Curing

**Figure 6:**
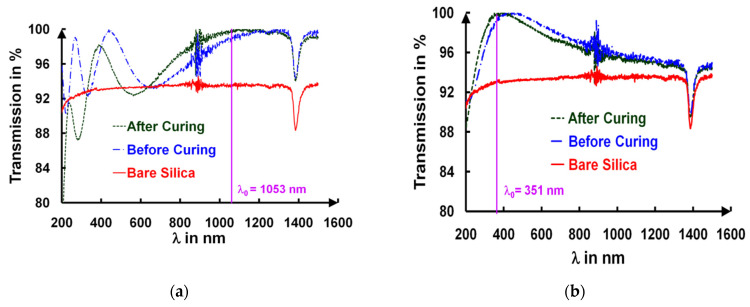
Spectral evolution of UV/visible of an AR 1ω coating (**a**) and an AR 3ω coating (**b**) before and after 17 h of curing.

The curing produced a reduction in the total layer thickness evidenced by UV/visible spectroscopy (cf. **Figure 6**) and estimated from a full fit of the spectrum that also gave the refractive index n_L_(λ) and the extinction coefficient k_L_(λ) based on Cauchy’s model, according to their wavelength dependences (see hereafter).

In **Figure 6**, the UV/visible spectra of an uncured and a cured layer are shown. **Figure 6a,b** correspond to an AR 1ω (t = 216 nm) and an AR 3ω (t = 72 nm) coating, respectively. The spectral transmission curves of the bare silica substrate are also given in **Figure 1** (continuous red curve). The spectral transmissions showed Fresnel’s interferences whose number of fringes increased with the thickness of the films, as expected. The separation between the two extrema of the transmission value corresponded to a difference in the optical path length of p.λ_0_/4, where p was the interference order and λ_0_ was the optical wavelength. As shown in **Figure 1a** (AR 1ω sample), five maxima, found at the wavelength positions λ = 1111 nm, 562 nm, 387 nm, 280 nm, and 266 nm, were reported over the spectral range [200–1200 nm] for a sample submitted to a curing time of 17 h. As shown in **Figure 1** only one extremum at λ = 373 nm was observed for the thinner AR 3ω film with the same curing conditions. It is worth mentioning that after 17 h of ammonia curing, a transmission decrease was also observed for the thicker films at a wavelength smaller than 700 nm (see **Figure 6a**). The minima positions exhibited a blue shift after the curing, which was consistent with the decrease in the layer thickness, i.e., a curing-induced shrinkage. The targeted antireflective properties were perfectly obtained for both the AR 1ω (**Figure 6a**) and AR 3ω (**Figure 6b**) samples from the curing process, reaching an almost 100% transmission that had to be compared to the value of 93.5% transmission of bare silica substrates.

**Figure 7:**
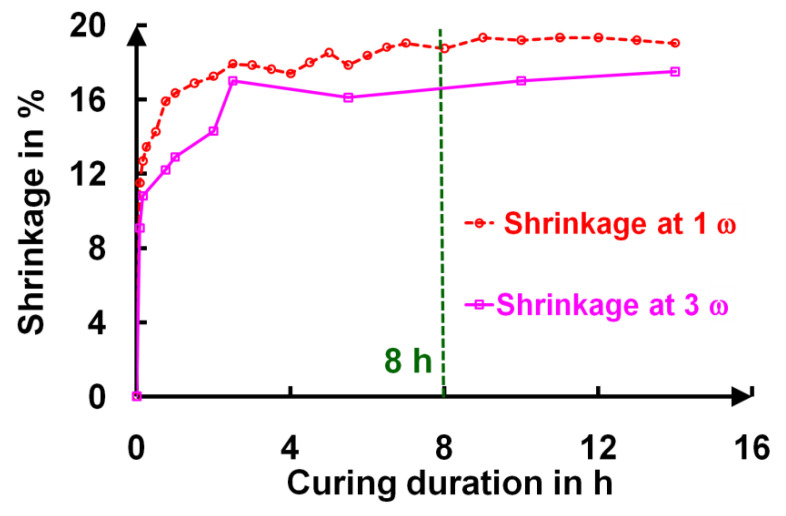
Shrinkage evolution of AR 1ω and AR 3ω as a function of the curing time.

The transition from an uncured to a cured layer was systematically followed as a function of the curing time. These transmission spectra were fit following a specific procedure [15]. These different fits provided the values of the thickness t as well as those of the refractive index n_L_(λ) and its extinction coefficient k_L_(λ) according to different curing times. For this fit, we used Cauchy’s law for the wavelength dependence of n_L_(λ) and k_L_(λ) The shrinkage amplitude is shown in **Figure 7**, where an asymptotic behavior was obtained after 8 h of curing, indicating the end of the chemical curing process. The shrinkage was slightly smaller for the AR 3ω film (17%) than for the AR 1ω (19%) film.

**Figure 8:**
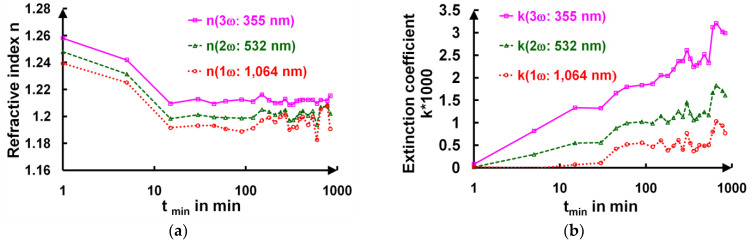
Evolutions of the refractive index n_L_ (**a**) and extinction coefficient k_L_ (**b**) calculated at three wavelengths (in nm) as a function of the curing time (t_min_: time in minutes) for the AR 1ω film.

Simultaneously to the decrease in the film thickness, the refractive index n_L_ (1ω: 1064 nm) of the AR 1ω film slightly decreased from 1.239 to 1.194 after 8 h of curing (**Figure 8a**). On the contrary, the extinction coefficient k_L_(1ω: 1064 nm) (**Figure 8b**) increased up to 1 × 10^−3^, and this was mainly attributed to the appearance of the crazing.

**Figure 9:**
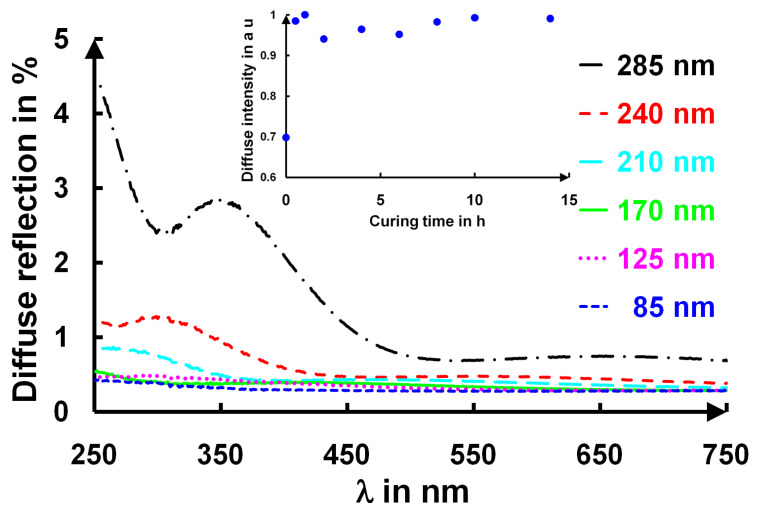
Diffuse reflection of silica coatings with different initial thicknesses after 17 h of curing and in the inset the diffuse intensity as a function of curing time for a 285 nm thick film.

We indeed noted that within this spectral range, the refractive index of the deposited film, n_L_, was always smaller (1.26) than the substrate’s refractive index, n_S_ (>1.44), and that the transmission coefficient value for a half-wave layer was smaller that for the substrates alone. This means that the cured layers became somehow more “absorbent” or rather more diffuse (**Figure 9**). The thermal extinction coefficients of these layers were in the 10^−6^ range [35]. For the AR 3ω sample, the crazing phenomenon did not occur because the layer thicknesses were thinner [16]. Moreover, the variations in the refractive index were invisible, as they were hidden in the inaccuracies of the measurements and fits.

**Figure 10:**
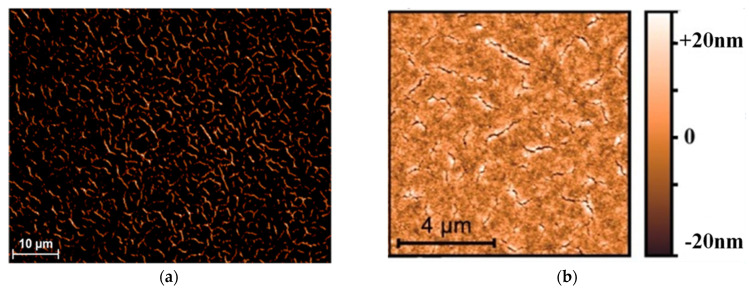
Example of crazing after ammonia curing seen through a dark field microscope (**a**) and measured by AFM (**b**).

**Figure 9** shows the diffuse reflection of several deposited films with different thicknesses for a curing period of 17 h. There was a clear increase in this diffuse reflection for the films thicker than 210 nm. This was in accordance with our different microscopy results presented in **Figure 10**. In the inset of **Figure 9**, for a layer of the same thickness, the diffuse intensity was independent of the curing time when the sample was submitted to an ammonia atmosphere.

This effect was attributed to the appearance of the crazing revealed by the optical microscopy and AFM [16] (**Figure 10**). The refractive indexes of the dry films were as low as 1.21, and the open porosity was estimated at around 55% in volume. These were calculated from the Lorentz oscillator model in the case of the two air/silica dipoles. The moderate hydrophobic character of the deposited layers (water wetting angles > 90°) efficiently protected the nanoparticle layers from water capillary condensation at ambient relative humidity. In the first minutes of curing, a slight variation in the refractive index was observed, which was followed by its stabilization.

### 2.3. Visualization of the Crazing after Ammonia Curing

Typical optical microscopy (**Figure 10a**) and AFM (**Figure 10b**) images of the coating revealed the multiscale network of the crazing, ranging from the nanometer scale up to the micrometer length.

**Figure 11:**
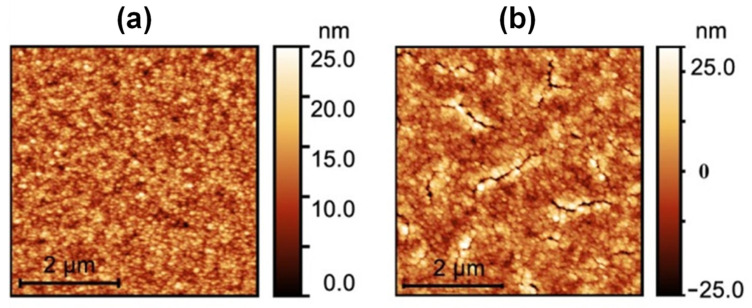
AFM image of a 210 nm thick (AR 1ω) film before (**a**) and after (**b**) 17 h of ammonia curing.

The obtained AFM images in a 5 × 5 µm^2^ area are given in **Figure 11** for a 210 nm thick film ((a) before curing and (b) after 17 h of curing). The uncured film showed a certain roughness, while the 17-h-cured film showed a cracked surface.

**Figure 12:**
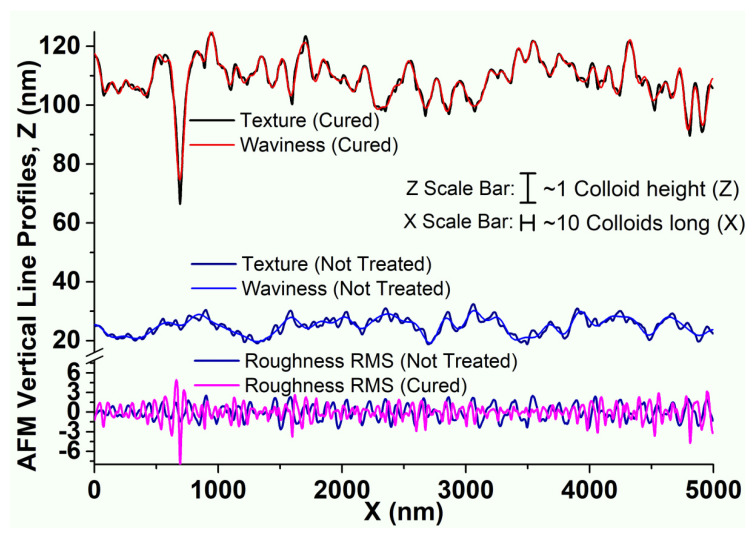
AFM profiles of a 210 nm thick (AR 1ω) film cured and not treated with texture, waviness, and roughness.

The profiles were taken for each image with a roughness analysis and are shown in **Figure 12**.

**Figure 12** shows the two profiles of the previous AFM images, where a crack and the roughness of the surface before and after crazing can be observed. The crack depth could reach 50 nm (which corresponded to four or five colloids), and their width could be close to 200 nm (twenty colloids). The roughness of the both surfaces was similar (R_q_ = 0.93 nm for an uncured layer and R_q_ = 0.90 nm for the cured layer), but the spatial frequency was twice as high for the cured film (9 µm^−1^) than for the uncured one (15 µm^−1^). Therefore, the nanoparticles could move during the ammonia curing. This demonstrates unambiguously that the stress due to the film contraction and/or chemical ripening promoted a local rearrangement of particles during the ammonia curing and an increase in the short-range order. This process has already been described in the chapter covering the analysis of the chemical bond modifications within the silica nanoparticle network.

Despite the appearance of crazing with the ammonia curing when the films were thick, the LIDTs were never affected regardless of the duration of the ammonia curing [14].

### 2.4. Mechanical Modifications Produced by Ammonia Curing

The transformation of the chemical bonds from Van der Waals interactions to hydrogen and covalent bonding was established here by IR spectroscopy (**Figure 2**), as discussed above. A direct consequence of this nanocontact chemical curing was a profound modification in the mechanical properties of the coating, which was associated with a strong modification of the nanoparticle surface interactions.

**Figure 13:**
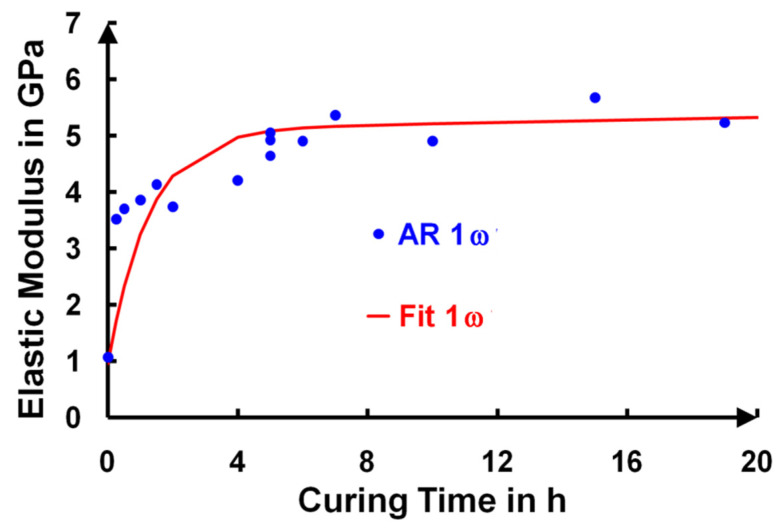
Determination of the elastic modulus (GPa) versus the curing time for AR 1ω layers.

A non-destructive and non-contact method was used to probe the mechanical properties of the coatings and was based on an all-optical laser ultrasonic technique. For a typical film thickness of 210 nm, we reported an increase in the elastic modulus from 1 GPa up to more than 5 GPa by increasing the curing time (**Figure 13**).

Let us recall that the bare silica modulus was around 40–60 GPa, which, nonetheless, depended on the structure considered. Following the nanocontact model previously discussed [21], these elastic modulus values made it possible to extract the nanocontact stiffness (“spring” stiffness between two nanoparticles) that ranged from 30 N·m^−1^ up to 150 N·m^−1^, which corresponded, according to the Derjaguin-Muller-Toporov (DMT) model [36], to a nanoparticle surface energy change that ranged from around 2 mJ·m^−2^ up to around 60 mJ·m^−2^. The drastic change in the surface energy we measured was also confirmed by macroscopic contact angle measurements [15].

### 2.5. Surface Modifications during Ammonia Curing

**Figure 14:**
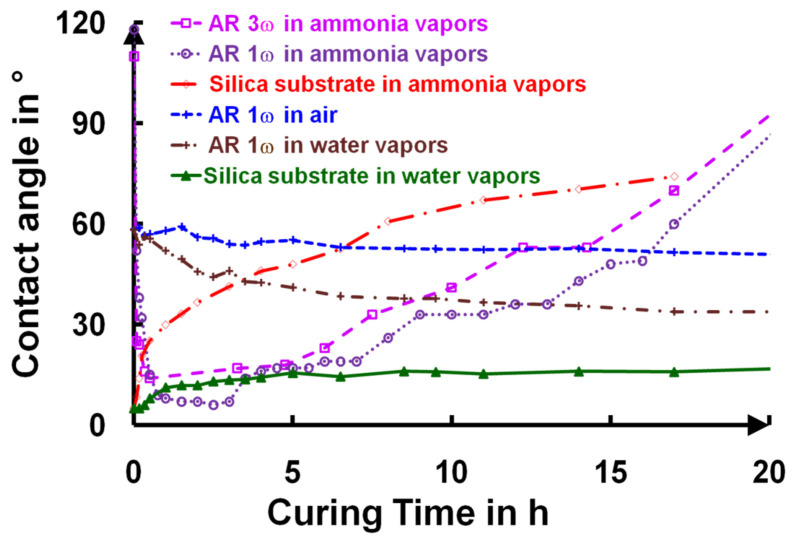
Change in the water-drop angle as a function of NH_3_ curing times for two different thicknesses and water vapor exposure times. All presented measurements were conducted in air at room temperature (T = 20 °C ± 0.5 °C) and controlled humidity (RH = 45% ± 5%).

The top surface layer modifications were also investigated by measuring the water-drop contact angle on the thin films (**Figure 14**) using a Krüss^TM^ instrument (Hamburg, Germany).

The deposited films presented a hydrophobic character (water wetting angle > 90°), with a water-drop contact angle of 110° due to the presence of unhydrolyzed Si–O–CH_2_CH_3_ groups. This moderate hydrophobic characteristic of the deposited layers efficiently protected the sol–gel layers from water capillary condensation at ambient relative humidity. In the first minutes of curing, a slight variation in the refractive index was observed (**Figure 8a**), which was caused by the disappearance of CH_2_ and CH_3_ and was followed by its stabilization. This state corresponded to a highly hydrophilic character appearing through the formation of Si–OH groups from the hydrolysis step. After five hours, the ammonia induced condensation of the silanols, which formed Si–O–Si bridges between the nanoparticles. This reaction then induced a slow increase in the contact angle. For example, for the AR 1ω and AR 3ω samples, during a twenty-hour curing time in ammonia vapor, the water contact angle gradually increased, and the surfaces of these AR coatings became hydrophobic (θ > 90°), while the layers were mechanically stable (**Figure 7** and **Figure 13**).

A similar increase in the hydrophobicity was observed on the silica substrates exposed to ammonia vapor, but this phenomenon did not occur in the AR 1ω sample when the silica substrates were exposed to air or water vapor; the contact angle remained stable over time.

**Figure 15:**
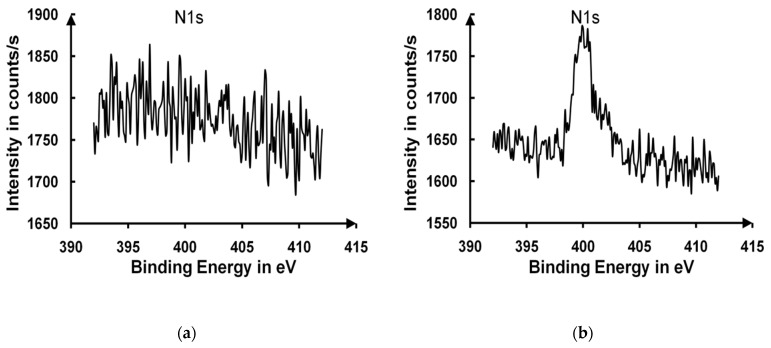
XPS signals of an uncured sample (**a**) and a cured sample exposed for 24 h to ammonia vapor (**b**).

Two assumptions were put forward concerning the high water contact angle obtained after the long curing times. The first hypothesis was to suspect the ammonia-related pollution of the surface layer (adsorption of ammonia at the surface of the particles) [37,38]. The second one was based on a drastic change in the top layer roughness that could explain the constant increase in the contact angle. Infrared spectroscopy measurements of transmission and reflection were unsuccessful in identifying any absorption peaks of the ammonia molecules, which were hidden by the stretching vibration bond, ν_O–H_(3000–3700 cm^−1^). XPS measurements have been performed on an uncured sample (**Figure 15a**) and a sample cured for 24 h (**Figure 15b**), and they only showed traces of nitrogen in the sample cured for 24 h.

## 3. Conclusions

The paper reported a detailed investigation of post-treatment ammonia curing at room temperature that is currently being used in the industrial production of large-scale optical components for the Laser MégaJoule (LMJ) set-up, which is composed of thousands of antireflective optical layers. The process has greatly improved the optical, mechanical and adhesion properties [21] that were tested in terms of laser damage (not shown here [14]). The treatment successfully minimized the longitudinal stress of the nanoparticle stacks without altering the final optical properties. The main disadvantage of this post-treatment was that it lasted 17 h and therefore was expensive.

By using a large set of spectroscopic and analytical methodologies, we optimized the time of this post-treatment (8 h against 17 h previously) while maintaining the mechanical properties (increased by a factor five in comparison with the uncured layers) and aiming for the desired properties (controlled shrinkage to achieve cured antireflective layers at the working wavelength, i.e., 351 nm or 1053 nm). Moreover, this process is of paramount importance because it can allow the perfect control of the thicknesses of the final silica films and it can facilitate the handling and maintenance conditions criteria.

The ammonia vapor curing treatment of the silica nanoparticle coatings greatly improved the robustness of the optical components developed for the LMJ as well as their manufacturing and maintenance costs. By selecting the initially deposited thickness and the optimal curing conditions, the route described here was particularly efficient for controlling the final optical and physical properties of the antireflective coatings that could be achieved on large-size optics and under industrial production requirements.

## 4. Materials and Methods

### 4.1. Chemical Synthesis of the Sol–Gel Solution

**Figure 16:**
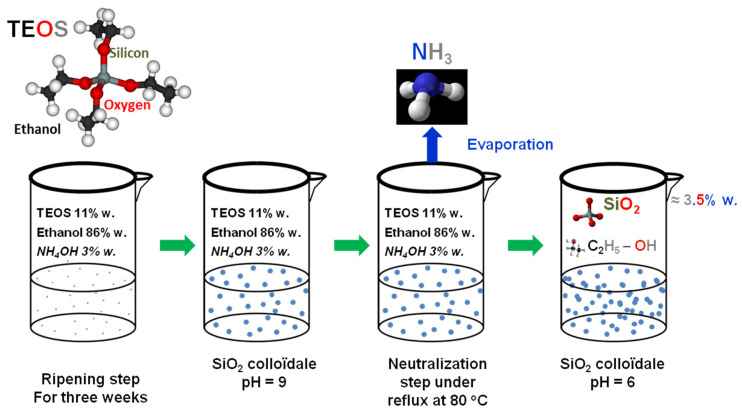
Procedure for colloidal silica solution preparation.

All nanoparticle films were deposited on optical components by dip-coating [39] from a sol–gel solution in an ISO 6 laboratory on polished silica samples or silicon wafers (50 mm in diameter). Thus, both substrate faces were coated at the same time. The sol–gel solution used for the antireflective treatment was a colloidal silica suspension synthesized by Stöber’s sol–gel method [7]. The synthesis is the result of hydrolysis–condensation in a basic medium (NH_4_OH) of tetraethyl orthosilicate (TEOS) in the solvent ethanol (see **Figure 16**).

### 4.2. Deposition Method

Films were deposited by dip-coating. Catalyzed silica sol was prepared by mixing a solution of EtOH, TEOS, and NH_3_·H_2_O. The molar ratio of TEOS:H_2_O:EtOH:NH_3_ was 1:2.2:3.4:1. The mixtures were stirred for 15 min at 25 °C and aged at 25 °C for 21 days. Then, they were refluxed until the pH reached 6–7. 

**Figure 17:**
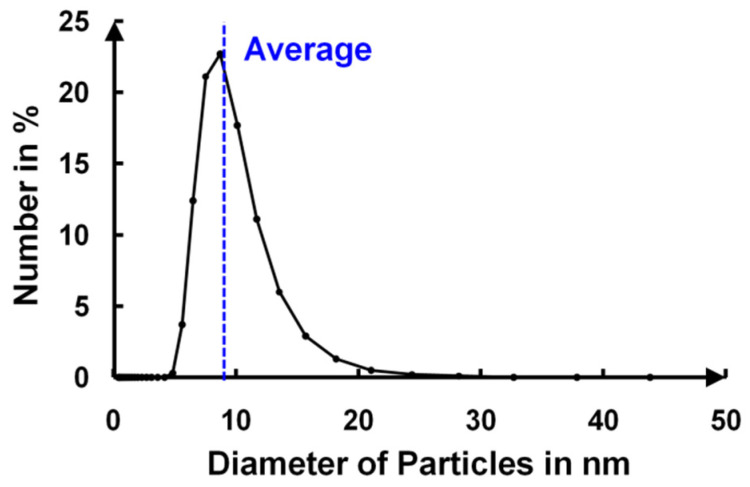
Size distribution of silica particles in nm.

Silica sol–gel solutions were studied using dynamic light scattering (DLS) experiments (on a Malvern Zetasizer Nano ZS device, Worcestershire, United Kingdom) to obtain the size distribution of the particles in a 0.5% ethanol solution. **Figure 17** shows the typical particle size distribution obtained from 5 replicates of the analysis. The final sol–gel solution contained particles with an average diameter of 10 nm. The deposited film was thus composed of a collection of 10 nm silica nanoparticles.

The withdrawal speed of the dip-coating was 0.15 m·min^−1^.

Two different film thicknesses, 210 nm and 70 nm, called AR 1ω and AR 3ω respectively, were investigated. They corresponded to antireflective coatings for 1053 nm (AR 1ω) and for 351 nm (AR 3ω), which are the two characteristic wavelengths of the LMJ [4].

### 4.3. Ammonia Curing

The cohesion between the particles and their adherence on the substrate were relatively weak, leading to a very low abrasive resistance of coatings. To increase the cohesion of the colloidal thin films, a chemical modification of the nanoparticle surfaces was achieved by a post-process using ammonia vapor, called the “ammonia curing process” [40]. The as-deposited AR-coating is kept for a certain time in a confinement chamber with alkaline vapors (vapor pressure of ammonia was 9 atm at 20 °C) obtained from concentrated an ammonium hydroxide aqueous solution (28% ammonia by weight) at standard pressure and temperature conditions, which were withdrawn to achieve the laboratory atmosphere again.

This process led to a change in the nanocontact chemical bonds from Van der Waals (VdW) to hydrogen and covalent bonds, inducing a significant increase in the elastic modulus [15,20,41,42].

We aimed to follow the chemical modifications brought by the ammonia curing process to understand this process and to improve it in the future. Different characterization techniques were used, such as IR spectroscopy, surface tension measurements (chemical changes inside the layer or on the surface), ultraviolet and visible spectroscopy (refractive index, extinction coefficient, and thickness checking), microscopy, and a picosecond acoustic method [15,20] (mechanical evolution).

### 4.4. Infrared Analysis

The surface chemistry of the coatings as a function of curing times was investigated by IR spectroscopy analysis of the characteristic vibrations of chemical bonds. Transmission measurements at different curing times using an FT-IR spectrophotometer (Spectrum Two FT-IR from Perkin Elmer Company (Waltham, MA, USA) over 400–4000 cm^−1^ with a 4 cm^−1^ resolution) were carried out at a 0° angle of incidence. Each spectrum was the result of 15 scans. The film thickness was set to 300 nm to enhance the IR signal on both sides of the polished silicon wafers. They were measured during the curing at given times to monitor the evolution of the chemical layer during curing.

### 4.5. Shrinkage of Layers after Ammonia Curing and Crazing

The chemical modifications of the particles and the reduction in the layer thickness were studied as a function of increasing the curing time. The variations in the thicknesses of the AR 1ω and AR 3ω sample were examined by UV/visible spectroscopy in the transmission mode (Perkin Elmer 900 spectrophotometer Waltham, Massachusetts, USA) over the 200–1500 nm spectral range using an integrated sphere, a scan rate of 600 nm·min^−1^, an average time of 0.1 s, and a 2 mm slit width. A zero and a baseline correction were recorded before all the measurements. The thickness measurement and the refractive index of the deposited and cured layers were obtained from Fresnel’s interferences and a comparison with a bare silica substrate following a standard method. The thicknesses, t_Not cured_ and t_Cured_, and the refractive indexes of the cured and non-cured layers were determined [15]. Thickness variations induced by the curing process were expressed as a percentage of the shrinkage in comparison with the initial thickness (t_Not cured_), using the following **Equation (3)**:

(3) $$\mathrm{shrinkage}=100\times \frac{t_{\mathrm{Not}\;\mathrm{cured}}-t_{\mathrm{Cured}}}{t_{\mathrm{Not}\;\mathrm{cured}\;}}$$

Thicknesses were optimized for the antireflective function at the targeted wavelength.

Each spectral curve was treated with the same fitting procedure to determine the refractive index n(λ), the extinction coefficient k(λ), and the thickness, and therefore the overall shrinkage was determined. This analysis of the transmission spectra of the films at 1ω and 3ω was conducted by the method of envelopes to determine the departure points and a fitting by the Newton–Raphson method explained in [15,43,44]. The calculations of the thickness from the curves allowed the determination of the layer shrinkage.

### 4.6. Crazing Detection

In some cases, the layer shrinkage after the NH_3_ curing generated micro-cracking at the top of the sample surface [45]. This crazing was observed by microscopy measurements (Leica DRM microscope (Wetzlar, germany) using a magnification ranging from ×10 to ×150 with an x & y motorized scanning stage and a digital camera DFW-V500 (Sony, Park Ridge, NJ, USA) in the dark-field mode [16]).

The possible change in the surface roughness after the curing process was investigated using AFM (AFM in the non-contact mode from Nano-World Innovative Technologies (Neuchâtel, Switzerland) using an NanoWorld Pointprobe® NCHR AFM probe with a typical spring constant of 42 N·m^−1^, a typically 320 kHz resonance frequency, and radius below 10 nm) [37,46].

The crazing of the layers generated some scattering and induced an increase in the extinction coefficient, k_L_, leading to a diffuse reflection increase in the ultraviolet and visible range that was measured with a Cary 5000 spectrophotometer using an integrating sphere at a 0° angle of incidence. The spectrophotometer was a double-beam spectrophotometer with a 200–2500 nm spectral range, a 600 nm·min^−1^ scan rate, a 0.1 s average time, and a 2 mm slit width. A zero and a baseline correction were conducted before the measurements.

### 4.7. The Measurement of Mechanical Properties

**Figure 18:**
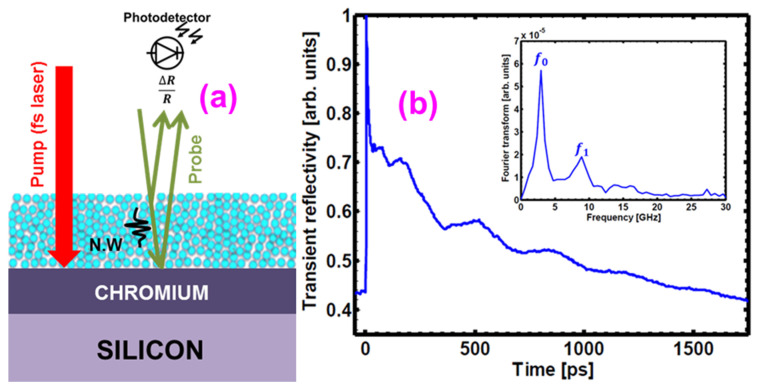
(**a**) Scheme of laser ultrasonic measurements carried out, where NW stands for nanoacoustic waves. An fs pump laser beam excited the sample leading to the generation of an acoustic wave in the chromium transducer. The acoustic wave propagated into the film under study and induced a local refractive index change in the material. The fs probe laser beam met this refractive index modification, and its reflection R of the sample was modified. This ΔR/R signal was detected (**b**) Transient optical reflectivity (ΔR/R) signal revealing the oscillating component due to the mechanical resonances of the coating.

The laser picosecond ultrasonic technique was employed to quantify the role of the curing process on the mechanical properties of the silica layers. This technique is an all-optical technique based on ultrashort laser pulses used in a pump-probe time-resolved configuration. It permitted the excitation and detection of mechanical vibrations of very thin layers and the extraction of the sound velocity at the nanoscale and thus evaluated the nanocontact strength [15,21,23,47]. It consisted of an excitation of an absorbing substrate (here, a metallic chromium layer was used as a transducer) with a laser pulse (pump pulse), which gave rise to the generation of nanoacoustic waves (**Figure 18a**). These nanometric waves (NW) penetrated the coating and induced mechanical resonances in the coating. A second femtosecond laser pulse (probe beam) allowed the detection of the changes induced by the NW in the studied film. Its optical reflectivity variation (∆R) was directly affected by these mechanical oscillations, giving the oscillatory components on the probe’s optical reflectivity function (∆R/R), as shown in **Figure 18b** [14,20,47]. These resonance frequencies followed an "organ-pipe" resonance sequence with f_p_, as given by **Equation (4)**.

(4) $$f_{p}=\left(2\times \;p+1\right)\times \frac{V}{4\times \;t}$$

where V is the sound speed, t is the thickness of the coating, and p is the order of the resonance. The elastic modulus, M, is finally obtained from **Equation (5)**.

(5) $$M=\rho \;\times {\;V}^{2}$$

where ρ is the volumic mass of the coating.

### 4.8. Contact Angle and XPS Measurements

Water-drop contact angle measurements were also performed on the same films (with a KrüssGBX apparatus with deionized water) submitted to variable curing times. Wetting measurements were taken 1.5 s after the deposition of the drop on a triplicate base and with a 1° precision. The chemical composition of the surface was analyzed by photoelectronic X-ray spectroscopy (XPS) to determine whether the cause of the increase in the angle of contact relative to the curing time of the hardened layers was not linked to a pollutant introduced during the curing process [38,48]. So, the chemical composition of the surface was analyzed. The XPS measurements were taken with an Omicron Argus X-ray photoelectron spectrometer (Omicron NanoTechnology GmbH, Taunusstein, Germany) with a monochromated AlK_α_ (hν = 1486.6 eV) radiation source with a 300 W electron beam power. The emission of photoelectrons from the sample was analyzed at a takeoff angle of 45° under ultra-high vacuum conditions (1 × 10^−8^ Pa). The XP spectra were collected at pass energy of 20 eV for the C1s, N1s, O1s, and Si2p core XPS levels. After the data collection, the binding energies were calibrated for the binding energy of the O1s peak at 532.5 eV. The peak areas were determined after the subtraction of a Shirley-type background. The atomic ratio calculations were performed after normalization using Scofield factors. The spectrum processing was carried out using the version 2.3.15 Rev 1.2 of the Casa XPS software package.

## Figures and Tables

**Table 1:** Summary of the difference between a free fit and a fit taking into account the bonding value determined by Innocenzi (see **Equations (1)** and (**2)**).

Curing Time in Hour	Model with Six Fitted Gaussian Curves				Model with Six Gaussian Curves According to Innocenzi			
	σ_i_	ω_i_	A_6G_	R^2^	σ_i_	ω_i_	A_Innocenzi_	R^2^
	cm^−1^	cm^−1^	a.u.	a.u.	cm^−1^	cm^−1^	a.u.	a.u.
0 h	1065	19	0.446	0.0003	1068	23	0.622	1.045
	1100	26	0.756		1120	33	0.741	
	1112	26	0.129		1180	14	0.231	
	1163	17	0.352		1151	11	0.000	
	1196	8	0.035		1210	14	0.150	
	1212	27	0.291		1232	32	0.212	
17 h	1050	18	0.485	0.0016	1068	25	0.776	0.009
	1076	17	0.647		1120	41	0.225	
	1108	26	0.304		1151	65	0.250	
	1168	44	0.426		1180	32	0.100	
	1219	19	0.082		1210	20	0.102	
	1223	10	0.010		1232	13	0.040	
